# A Case Report and Literature Review of Intestinal Perforation Due to Tuberculosis

**DOI:** 10.7759/cureus.43241

**Published:** 2023-08-09

**Authors:** Angie E Vargas Rodríguez, Ansony R Godinez Vidal, Raymundo Alcántara Gordillo, Cristobal S Duarte Regalado, Jesús O Soto Llanes

**Affiliations:** 1 Surgery, General Hospital of Mexico Dr. Eduardo Liceaga, Mexico City, MEX; 2 Colorectal Surgery, General Hospital of Mexico Dr. Eduardo Liceaga, Mexico City, MEX

**Keywords:** acute presentation, abdominal pain, perforation, intestinal, extrapulmonary tuberculosis

## Abstract

Intestinal tuberculosis (ITB) is challenging due to its nonspecific clinical presentation, sometimes manifesting with acute complications such as intestinal perforation or obstruction.

We present the case of a 39-year-old male presented with continuous fever, abdominal pain, and peritoneal irritation. A contrast-enhanced thoracoabdominopelvic computed tomography revealed free air and fluid, suggestive of intestinal perforation. Urgent surgical treatment was performed via exploratory laparotomy, including right hemicolectomy and construction of a Brooke ileostomy. Histopathological analysis confirmed intestinal miliary tuberculosis.

The most commonly affected areas in ITB are the ileocecal region and ileum. Symptoms include abdominal pain, weight loss, changes in bowel habits, and fever. Contrast-enhanced computed tomography is crucial for diagnosis. The first-line treatment is medical with antituberculosis drugs.

Due to its delayed diagnosis, ITB should be considered in patients with nonspecific and progressive symptoms. Early medical management is crucial to prevent acute complications associated with high morbidity and mortality.

## Introduction

Tuberculosis (TB) remains a globally relevant infectious disease, with an estimated 10.6 million people falling ill with TB worldwide in 2021, resulting in 1.6 million deaths, including 187,000 individuals with human immunodeficiency virus (HIV) co-infection [1]. TB is the second most deadly infectious disease after coronavirus disease 2019 [1]. Mexico is considered an endemic country for TB, with over 28,000 cases of TB reported annually, and 30% of carriers unaware of their condition [2].

TB can affect any part of the gastrointestinal tract, from the oral cavity to the anus, including the peritoneum, mesenteric lymph nodes, and solid organs such as the liver, spleen, kidney, or adrenal glands [3]. The incidence of extrapulmonary tuberculosis (EPTB) has been increasing, accounting for one in five TB cases, likely due to the concomitant rise in HIV infection [1]. Intestinal tuberculosis (ITB) represents 2% of TB cases worldwide and ranks sixth among EPTB cases [4,5]. The clinical manifestations are often nonspecific and can mimic other pathologies, with inflammatory bowel disease or malignant neoplasms being the main differential diagnoses. Misdiagnosis occurs in 50-70% of cases [5], leading to increased morbidity and mortality. While first-line treatment is medical with antituberculosis drugs, urgent surgical intervention is necessary when acute complications such as obstruction or perforation arise. We present a case of intestinal perforation secondary to ITB in a patient in his fourth decade of life with no history of previous contact with TB patients.

## Case presentation

A 39-year-old male, born and raised in Mexico City, with a history of psychomotor retardation and open cholecystectomy for acute calculous cholecystitis in 2020, presented to the emergency department with a two-year clinical history of postprandial abdominal pain, one to two episodes of diarrhea per week, a weight loss of 25 kg over two years, occasional fever up to 39°C, nonproductive cough, and dyspnea. Two days before visiting the emergency department, he experienced an increase in the intensity of abdominal pain, decreased appetite, continuous fever, asthenia, and adynamia.

**Figure 1:**
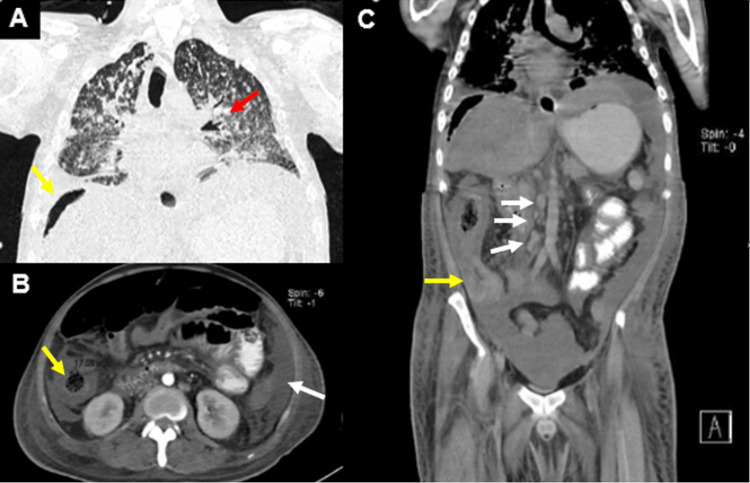
Computed tomography scan. (A) Coronal section. Randomly distributed nodular consolidation areas throughout the lung parenchyma, predominantly peribronchovascular, and consolidation involving almost the entire lower lobes with air bronchograms (red arrow). Subdiaphragmatic free air (yellow arrow). (B)Axial section. Concentric thickening of the ascending colon (yellow arrow) and free fluid in the left paracolic gutter (white arrow). (C)Coronal section. Abundant homogeneous air and fluid in the cavity, thickening of the walls of the distal ileum up to 11.3 mm, cecum, and ascending colon with concentric wall thickening up to 17 mm, with heterogeneous enhancement upon contrast application (yellow arrow); inter-aortocaval lymph nodes with intense enhancement (white arrows).

**Figure 2:**
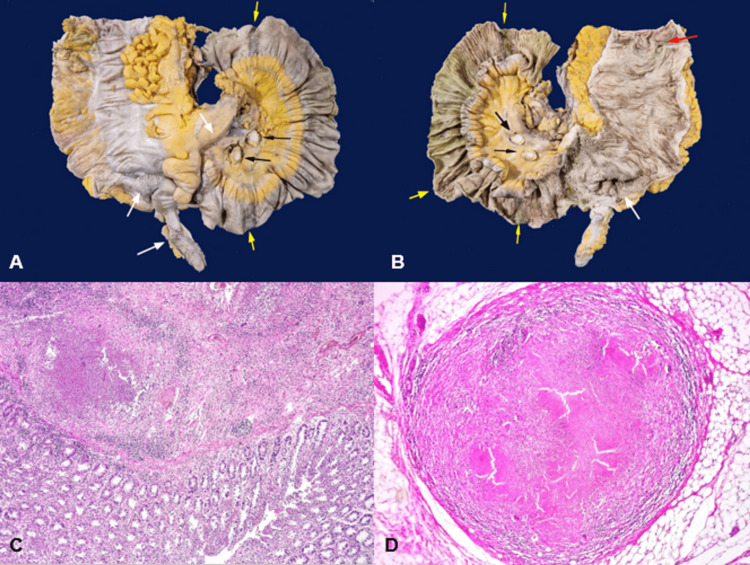
Pathological anatomy. (A) Serosal surface of the terminal ileum, cecal appendix, cecum, and ascending colon. Yellow arrows indicate areas perpendicular to the longitudinal axis of the intestine, dark brown in color, with multiple light brown pinpoint lesions (miliary pattern) in the terminal ileum. White arrows show multiple light brown pinpoint lesions (miliary pattern) in the cecal appendix, cecum, and mesentery. Black arrows indicate the cut surface of two mesenteric lymph nodes with caseous necrosis.(B) Mucosal surface of the terminal ileum, ileocecal valve, cecum, and ascending colon. Yellow arrows show ulcers perpendicular to the longitudinal axis of the intestine in the terminal ileum, associated with the serosal surface changes mentioned in A. The largest ulcer measures 2.0 cm in width and involves the entire intestinal circumference, while the smallest measures 1.0 × 0.3 cm. The white arrow shows an ulcer affecting the ileocecal valve and part of the cecum, measuring 7.5 × 7.0 cm. The red arrow shows a 2.5 cm × 1.0 cm ulcer in the ascending colon. (C) (hematoxylin and eosin (H&E) 40×) Colonic mucosa with abundant acute and chronic inflammatory infiltrate, and in the submucosa, a suppurative granuloma is identified, consisting of central necrosis, acute inflammatory infiltrate, epithelioid cells, some multinucleated giant cells, and lymphocytes at the periphery. (D) (H&E 40×) Tuberculoid granuloma. Well-circumscribed lesion showing central caseous necrosis, epithelioid cells, intercalated multinucleated giant cells, and lymphocytes at the periphery.

**Figure 3:**
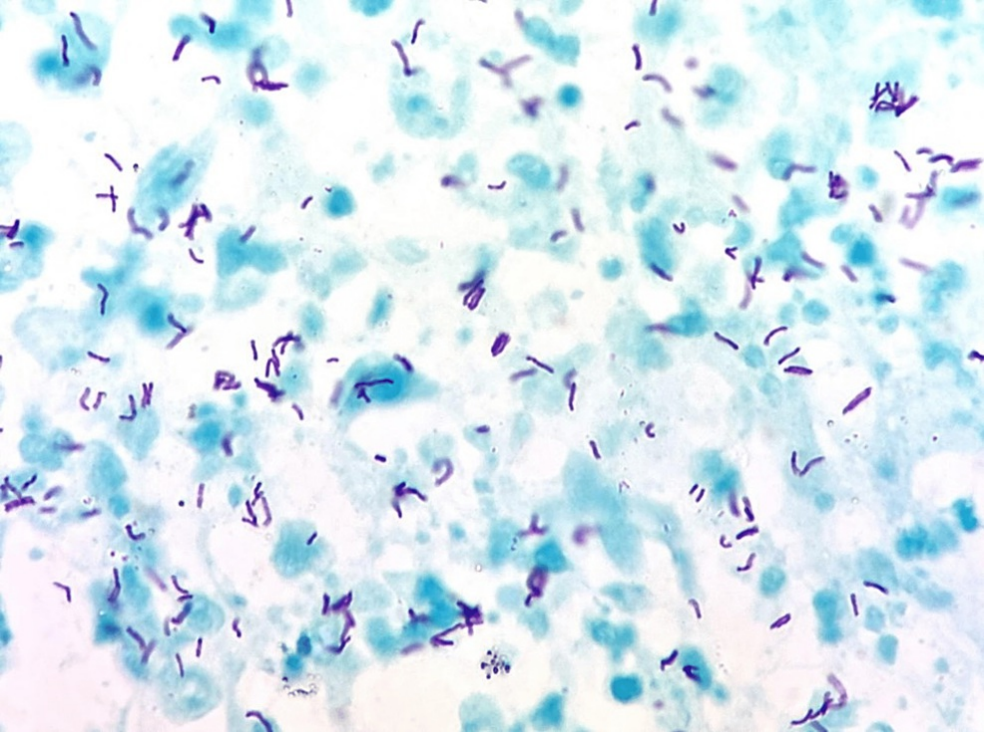
Ziehl-Neelsen staining. Ziehl-Neelsen staining is positive for abundant acid-alcohol-resistant bacilli (AFB) (1,000×).

On arrival at the emergency department, he exhibited signs of peritoneal irritation and bilateral crepitant rales on physical examination. Laboratory tests revealed bicytopenia, specifically leukopenia and thrombocytopenia, as well as electrolyte imbalance characterized by hyponatremia, hypomagnesemia, and hypoalbuminemia (Table 1). A contrast-enhanced thoracoabdominopelvic computed tomography (CT) was performed due to acute abdomen, revealing multiple hyperdense nodular lesions smaller than 3 mm in size scattered throughout the lung parenchyma, random peribronchovascular distribution of nodular consolidation throughout the lung parenchyma, and consolidation with air bronchograms in the lower lobes (Figure 1). In the abdomen and pelvis, abundant free homogeneous air and fluid were observed in the cavity, along with thickening of the distal ileum walls up to 11.3 mm, cecum and ascending colon with concentric wall thickening up to 17 mm, showing heterogeneous enhancement after contrast administration. The transverse, descending, and sigmoid colon were collapsed with wall thickening up to 7.9 mm. The retrocecal appendix exhibited an increased diameter of up to 12.8 mm with wall thickening (Figure 1). Multiple mesenteric and retroperitoneal lymph nodes were present at the para-aortic and inter-aortocaval levels, appearing rounded with regular and well-defined borders, compatible with disseminated TB with pulmonary, splenic, and intestinal involvement, along with pneumoperitoneum suggestive of intestinal perforation. Based on the CT findings, urgent surgical intervention was requested, and an exploratory laparotomy was performed, revealing two perforations of the distal ileum at 20 and 40 cm from the ileocecal valve, both measuring 0.3 × 0.3 cm on the antimesenteric border and multiple light brown pinpoint lesions (miliary pattern) measuring 2 × 2 cm in the largest, in the cecum and ascending colon, suggestive of ITB. A site of stenosis was found 40 cm from the ileocecal valve, and moderate ascites was present. Right hemicolectomy, resection of 70 cm of the distal ileum, and construction of a Brooke ileostomy were performed. A transoperative platelet apheresis transfusion and one unit of red blood cell concentrate transfusion were administered. The resected specimens were sent for pathological examination, which confirmed chronic granulomatous and acute abscessed inflammation due to miliary TB in the ileum, ileocecal valve, cecum, appendix, ascending colon, mesentery, mesoappendix, peritoneum, mesenteric lymph nodes, and mesenteric blood vessels. Eight ulcers were reported in the ileum mucosa, with the largest measuring 2 cm, the cecum with a 7.5 × 7 cm ulcer, and the ascending colon with a 2.5 × 1 cm ulcer (Figure 2). Ziehl-Neelsen staining was positive, revealing abundant acid-fast bacilli, confirming the diagnosis of ITB (Figure 3). After a two-week hospital stay with appropriate post-surgical progress and ruling out primary immunodeficiencies and HIV infection, the patient was evaluated by the infectious disease service with histopathology results. Antituberculosis treatment was initiated, leading to symptom improvement after three months, with resolution of fever, asthenia, and adynamia, as well as decreased episodes of abdominal pain and decreased appetite. Follow-up was conducted at an outpatient clinic 15 days after discharge and subsequently on a monthly basis.

**Table 1:** Laboratory results on admission.

Analyte	Patient’s finding	Normal range
Leucocytes	2.5	4.5–10 × 10^3^/µL
Neutrophils	95	40–70%
Lymphocytes	2.4	20–30%
Hemoglobin	9.5	12–16 g/dL
Platelets	97	150–450 × 10^3^/µL
Glucose	65	74–106 mg/dL
Urea	34	17–43 mg/dL
Creatinine	0.47	0.66–1.09 mg/dL
Sodium	123	136–145 mEq/L
Potassium	3.9	3.5–5 mEq/L
Chloride	95	98–107 mEq/L
Magnesium	2	1.9–2.5 mg/dL
Albumin	1.76	3.5–5.2 g/dL
Procalcitonin	3.3	<0.5 ng/mL
C-reactive protein	204	0–19 mg/dL

## Discussion

TB is an endemic infectious disease in Mexico, with an annual incidence of 15.7/100,000 inhabitants [6]. Although it primarily affects the lungs, it has been reported that up to 25% of cases involve extrapulmonary sites [7], including ITB, which ranks sixth among EPTB cases. ITB usually affects men in their third or fourth decade of life [5,8,9], which matches the age range of our patient. It is more common in populations where TB is endemic, as is the case in our country. It is important to mention that our patient did not have contact with TB patients and had a complete childhood vaccination history, with no other risk factors for immunosuppression and a negative HIV test.

*Mycobacterium tuberculosis* is the bacilli responsible for ITB. It can develop through primary infection or secondary reactivation from a primary lung focus, with the latter being the most common cause. Proposed routes of gastrointestinal infection include ingestion of the bacilli from sputum from an active lung focus, hematogenous dissemination from the lung, lymphatic dissemination (through M cells, found in the epithelium of Peyer’s patches, which provide an entry pathway for pathogens into the mucosa and can phagocytose TB bacilli), or direct spread from adjacent organs [4,10].

The main affected areas in ITB are the ileocecal region (44-84%) and the ileum (34%) [3,9]. Isolated involvement of the colon, perianal disease, or exclusive appendiceal disease has also been reported. The significant involvement of the ileocecal region is thought to be due to abundant lymphoid tissue and fecal stasis, which increase bacilli absorption and proximity to the mucosa [4]. Once the bacilli invade the submucosa, a granulomatous inflammatory response ensues, accompanied by vasculitis, thickening of the submucosa and serosa, and ulceration that can lead to perforation or fibrosis [4]. There are three forms of presentation, namely, ulcerative, hypertrophic, or ulcerohypertrophic or fibrous [10]. In this case, the patient presented predominant involvement of the ileum with eight ulcers and two perforations, as well as significant cecal involvement with a large ulcer and thickening of the appendix, with the ulcerohypertrophic form predominating.

Regarding the clinical presentation, the main symptoms include abdominal pain (74%), weight loss (59%), nausea or vomiting (31%), changes in bowel habits (25%), and fever (20%) [5,9]. Our patient experienced all of these symptoms over a two-year period. The main symptom was intermittent abdominal pain of mild to moderate intensity, typically postprandial. However, up to 2% of patients may present only with respiratory symptoms or constitutional symptoms and no abdominal symptoms [9]. As the clinical presentation is nonspecific, the time to diagnosis from the onset of symptoms is usually between three and 26 weeks [5], leading to delayed diagnosis and increased morbidity in cases of acute presentation manifesting as intestinal obstruction or perforation in unsuspected or undiagnosed TB.

Among the laboratory studies that can be conducted are the purified protein derivative skin test (PPD), which may yield positive results in 52-88% of cases [4]. Additionally, the interferon-gamma release assay, such as the T-spot test, measures the release of interferon-gamma by specific T-cells in response to in vitro stimulation with *Mycobacterium tuberculosis* antigens, and it shows positive results in 86% of patients with ITB [4]. It is essential to note that a negative result does not rule out the diagnosis. The latter test has demonstrated higher specificity compared to PPD in diagnosing latent or active TB infection and increased diagnostic sensitivity in populations with low TB incidence [3]. In our patient, these tests were not performed due to the unavailability of resources in the hospital and the patient’s limited financial resources.

Imaging studies are recommended to guide the diagnosis and play a crucial role as an adjunctive method. Contrast-enhanced CT can reveal intestinal wall thickening, intra-abdominal lymphadenopathy, intra-abdominal collections, thickened peritoneum, fistulas, and free or loculated ascites [11,12], which, along with the aforementioned nonspecific clinical features, can suggest intestinal TB. CT is the preferred modality as it can detect both extramural and intramural changes, as well as pulmonary TB or the involvement of other organs. In cases of acute presentation, such as our patient’s case, CT can provide evidence of intestinal perforation or obstruction, guiding the need for urgent surgical management.

Endoscopy is useful as an additional diagnostic tool or to confirm the diagnosis through biopsies for microbiological and histopathological analysis [4]. However, it has limited utility in cases of acute complications. Typical findings may include ileocecal inflammation, transverse or annular ileocecal ulcers, and ileal stenosis [9].

In the literature, first-line treatment for patients without acute complications is traditional antituberculous therapy for pulmonary TB for six to nine months [4,13]. Surgical treatment is reserved for patients who do not respond to medical treatment or those with acute complications such as intestinal obstruction, perforation, abscess, or fistulas [9]. The main indication for surgical treatment is intestinal obstruction due to stenosis [3]. In cases of intestinal perforation, the most common site is the antimesenteric border of the terminal ileum [14], and multiple perforations are more frequent in patients with HIV infection [15]. Symptomatic improvement with medical treatment is reported to be 99% at two to six months [3]. Our patient presented acutely with intestinal perforation, necessitating immediate surgical intervention. However, the patient also had associated pulmonary and splenic TB, leading to the initiation of antituberculous therapy following surgical treatment, which resulted in satisfactory improvement.

## Conclusions

ITB represents a diagnostic challenge that we encounter more frequently in developing countries due to its nonspecific clinical presentation. It should be considered as a differential diagnosis in patients with chronic abdominal pain, a history of HIV, any immunodeficiency, contact with TB patients, or even in patients without apparent previous contact, as in our case. With an early diagnosis, we will have the opportunity for timely initiation of antitubercular treatment, which is the first-line treatment, thereby avoiding acute complications such as intestinal perforation or obstruction, where urgent surgical intervention becomes crucial.
